# Impact of ALK inhibitors to potentiate ALK.CAR‐T therapy in neuroblastoma

**DOI:** 10.1002/ctm2.1732

**Published:** 2024-06-14

**Authors:** Elisa Bergaggio, Roberto Chiarle

**Affiliations:** ^1^ Department of Pathology Boston Children's Hospital and Harvard Medical School Boston Massachusetts USA; ^2^ Division of Hematopathology IEO European Institute of Oncology IRCCS Milan Italy; ^3^ Department of Molecular Biotechnology and Health Sciences University of Torino Torino Italy

1

Neuroblastoma is the most common extracranial solid tumour in children, representing about 7% of all malignancies under 15 years of age. Despite new treatment options, the median overall survival for high‐risk patients is around 50%, and overall survival for patients with relapsed/refractory disease is dismal. Thus, new drugs and therapeutic strategies are urgently needed.

Chimeric antigen receptor (CAR) T‐cell therapy is an immunotherapy that has revolutionised the field of oncology, leading to dramatic improvement in the treatment of haematologic malignancies. Unfortunately, this success has not been replicated in solid tumours so far despite a huge research activity in this field. Recent data on CAR‐T cells targeting GD2 in neuroblastoma patients demonstrated safety and remarkable tumour response.^1^ These results have created excitement for the use of CAR‐T cells against neuroblastoma and the importance of identifying optimal cell‐surface targets to increase CAR‐T‐cell efficacy.

Targeting anaplastic lymphoma kinase (ALK) has emerged as one of the most promising and specific approaches for neuroblastoma therapy. Most neuroblastomas express the full‐length ALK receptor,^2^, ^3^ and high expression of ALK correlates with poor prognosis.^4^ Activating mutations of full‐length ALK are found in 8%−12% of neuroblastomas, including familial neuroblastoma, and ALK amplification is found in 2%−3% of neuroblastomas, increasing the risk of relapse.^5^ Several studies have demonstrated that activated ALK is an oncogenic driver in neuroblastoma, especially when associated with MYCN amplification.^6^ These findings provide a rationale to target ALK in neuroblastoma. Recently, promising clinical responses have been observed with the third‐generation ALK inhibitor lorlatinib in patients with neuroblastoma carrying ALK genetic alterations.^7^ Also, antibody–drug conjugate directed to ALK demonstrated some efficacy against neuroblastoma in preclinical models.^2^ Initial approaches of targeting ALK with CAR‐T cells using the ALK48 scFv‐binding domain demonstrated limited preclinical efficacy owing to insufficient ALK target density.^8^ Recently, we demonstrated robust preclinical efficacy of an ALK‐CAR created with a different scFv binder.^3^ ALK.CAR‐Ts were as effective as GD2.CAR‐Ts against a large panel of neuroblastoma lines. In vivo, ALK.CAR‐T cells achieved 100% cure rate in a mouse model of human metastatic neuroblastoma with ALK amplification. In contrast, ALK.CAR‐Ts and GD2.CAR‐Ts were less potent against neuroblastoma tumours with lower ALK or GD2 expression, confirming the importance of antigen density for CAR‐T‐cell efficacy.

These findings raise the challenge of targeting tumours with low antigen expression. We discovered a mechanism to increase the surface display of the ALK target on tumour cells. Treatment of neuroblastoma cells with ALK inhibitors enhanced total and surface expression of ALK, especially in ALK‐mutated cells. The combination of lorlatinib and CAR‐T cells selectively potentiated the efficacy of ALK.CAR‐Ts but not of GD2.CAR‐Ts, supporting the specificity and synergy of this combination (Figure 1). Mutated ALK receptors are mainly intracellular due to defective glycosylation, but regain cell‐surface localisation upon kinase inactivation mediated by tyrosine kinase inhibitors (TKIs),^9^ enhancing susceptibility to ALK.CAR‐Ts. Notably, while an increase in ALK expression was clearly detectable in neuroblastoma with ALK mutations, the enhanced killing by ALK.CAR‐Ts was also observed in neuroblastoma with wild‐type ALK. Processing and regulation of wild‐type ALK remain unclear; however, TKIs can block the internalisation and degradation of the engaged receptor and block pathways involved in ALK transcription. Stratifying eligible neuroblastoma patients for ALK.CAR‐T therapy could use the ALK genetic status and expression levels as a biomarker; nonetheless, the majority of neuroblastoma patients could benefit from this combination therapy irrespective of their ALK genetic status.

**FIGURE 1:**
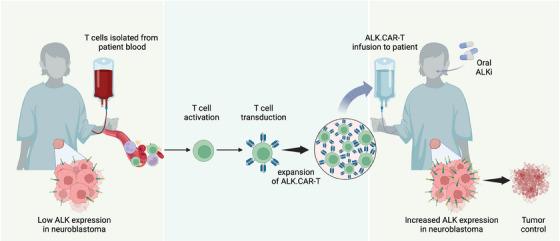
T cells are isolated from patients’ blood, activated, and transduced with a retrovirus to generate ALK.CAR‐T cells. The ALK.CAR‐Ts are expanded ex vivo and injected back into patients’ bloodstream. Administration of anaplastic lymphoma kinase inhibitors (ALKi) enhances ALK expression on the surface of neuroblastoma cells, resulting in an increased targeting by ALK.CAR‐T cells and tumour killing.

Although the combination of lorlatinib with ALK.CAR‐Ts enhanced anti‐tumour activity, neuroblastoma tumours with low ALK expression were not fully eradicated. These residual tumoural cells may represent a clonal expansion or a subset of cells with very low ALK expression in which lorlatinib does not induce sufficient increase of surface expression. Reacquisition of ALK expression upon ex vivo cultivation suggested that strategies such as intermittent ALK TKI administration, higher doses, or enhanced cell surface stabilisation may further improve the efficacy of ALK.CAR‐Ts.

Importantly, limited ALK expression in healthy tissues is fundamental to avoid toxicities by ALK.CAR‐T cell therapy. ALK expression in normal cells is restricted to developing organs in embryos and is believed to play a role in the regulation of neuronal differentiation during human embryogenesis. Analysis of mRNA expression profiles in an extended dataset of human tissues showed low levels of ALK transcripts in the hypothalamus, colon, pituitary gland, and testis.^3^, ^10^ However, comprehensive assessment of ALK expression in normal human tissues from healthy donors by immunohistochemistry showed low or undetectable ALK expression, apart from some staining in enteric neurons. The safety and specificity of ALK.CAR‐Ts were testable in mouse models due to the cross‐reactivity of ALK.CAR‐Ts against both human and mouse ALK. Consistent with ALK limited expression in normal tissues, we did not observe detectable toxicities of ALK.CAR‐Ts in mice. In keeping with this absence of toxicities, histology and immunohistochemistry for human CD3^+^ T cells in the hypothalamus and the enteric neurons in the intestinal wall did not show evidence of tissue damage or ALK.CAR‐Ts accumulation. However, increased infiltration of T cells was observed in the small and large intestines of mice treated with lorlatinib together with ALK.CAR‐Ts. These findings warrant for potential intestinal toxicity of lorlatinib‐ALK.CAR‐Ts co‐administration, but they also underscore the likely on‐target specificity of this approach. Importantly, the combination was well tolerated in mice. Similar intestinal infiltrates were observed in mice treated with GD2.CAR‐Ts, and intestinal toxicity is manageable in patients treated with GD2.CAR‐Ts. Therefore, there is suggestion that toxicity eventually arising with the combination of lorlatinib with ALK.CAR‐Ts could be managed in patients. Notably, the rapid reversal of ALK expression within 12 h upon discontinuation of ALK TKI and its subsequent reoccurrence with lorlatinib treatment provides the option to suspend the drug to normalise ALK levels. This suggests that implementing treatment cycles with lorlatinib interspersed with wash‐off periods could be a strategy to manage patients showing toxicity.

Overall, these preclinical data support the implementation of a phase 1 clinical trial to test ALK.CAR‐T cells in combination with lorlatinib in children with refractory/relapsed neuroblastoma. This trial will give us important information about the efficacy and safety of ALK.CAR‐T cells and their combination with ALK inhibitors. Moreover, eventual positive results could be translated to other tumours expressing ALK, thus expanding the number of patients who could benefit from this treatment.

## AUTHOR CONTRIBUTIONS

Elisa Bergaggio and Roberto Chiarle conceived and wrote the manuscript.

## DECLARATION OF INTERESTS

E.B. and R.C. filed a patent covering the development of ALK.CAR‐T cells.
